# Androgen Receptor and Tumor-Associated Neutrophil Expression Across Breast Cancer Subtypes: Associations With Clinicopathological Characteristics

**DOI:** 10.1155/ijbc/8209394

**Published:** 2025-07-22

**Authors:** Minoosh Moghimi, Shahin Vadoudi, Majid Amirian, Farzane Ahmadi, Mohammad Borna Amirian, Kasra Khodadadi, Reza Mansouri, Mahsa Mahjani, Sepehr Gohari

**Affiliations:** ^1^Department of Hematology-Oncology, Faculty of Medicine, Zanjan University of Medical Sciences, Zanjan, Iran; ^2^Department of Internal Medicine, Zanjan University of Medical Sciences, Zanjan, Iran; ^3^Borna Research and Medical Laboratory, Zanjan, Iran; ^4^Department of Biostatistics and Epidemiology, Faculty of Medicine, Zanjan University of Medical Sciences, Zanjan, Iran; ^5^Department of Gastroenterology, Tabriz University of Medical Sciences, Tabriz, Iran; ^6^Endocrine Research Center, School of Medicine, Shahid Beheshti University of Medical Sciences, Tehran, Iran; ^7^Student Research Center, School of Medicine, Zanjan University of Medical Sciences, Zanjan, Iran

**Keywords:** androgen receptor, breast cancer, cross-sectional study, estrogen receptor, pathology, tumor-associated neutrophil

## Abstract

**Objectives:** This study is aimed at evaluating androgen receptor (AR) and tumor-associated neutrophil (TAN) expressions in different breast cancer subtypes and their relationship with tumor differentiation, stage, and other clinicopathological markers.

**Methods:** A cross-sectional study was conducted on 84 breast cancer patients at Stages I–IV. Tumor tissues were assessed using immunohistochemistry for ER, PR, HER2, AR, and Ki67, along with TAN evaluation using hematoxylin and eosin staining. Associations between AR, TAN, and other clinical variables were analyzed using chi-square, *t*-tests, and logistic regression.

**Results:** AR was expressed in 70.2% of tumors and was significantly associated with ER positivity (OR = 74.31, *p* < 0.001), PR positivity (OR = 6.8, *p* = 0.01), and better differentiation (OR = 0.1 for poorly differentiated tumors, *p* = 0.035). AR positivity was highest in Luminal A/B subtypes (82%) and lowest in triple-negative breast cancer (TNBC) (20%; OR = 0.06, 95% CI: 0.01–0.3). In contrast, TAN positivity was observed in 45.6% of cases and was most frequent in TNBC (67%; OR = 3.7, 95% CI: 0.9–15.3) and poorly differentiated tumors (71.4%). TANs were inversely associated with PR positivity (OR = 0.21, *p* = 0.014) and showed a significant association with vascular invasion (*p* = 0.047). No significant associations were found between AR or TAN expression and metastatic status or neural invasion.

**Conclusion:** AR is a defining marker for HR-positive breast cancers and may serve as an indicator of lower tumor grade and differentiation status. TANs, however, are linked to more aggressive phenotypes, especially in TNBC, suggesting a role in driving tumor progression. This highlights the potential for AR and TAN expression patterns to refine patient stratification across breast cancer subtypes.

## 1. Introduction

Breast cancer is a complex and heterogeneous disease with distinct subtypes that exhibit varying clinical behaviors and responses to treatment [1]. Among these subtypes, hormone receptor-positive (HR+) and triple-negative breast cancer (TNBC) stand out due to their distinct molecular profiles and prognoses. HR+ cancers, which are characterized by the presence of estrogen (ER) and/or progesterone (PR) receptors, generally have a more favorable outcome and respond well to endocrine therapies [2]. Conversely, TNBC, defined by the absence of ER, PR, and HER2 (human epidermal growth factor receptor 2), is associated with a higher risk of recurrence, poorer prognosis, and limited therapeutic options [3].

One emerging area of research is the role of androgen receptor (AR) expression across breast cancer subtypes. AR, a member of the steroid hormone receptor (HR) family, is present in approximately 70%–80% of breast cancers but varies significantly depending on subtype [4]. In HR+ breast cancers, AR expression has been associated with less aggressive features [5]. However, its role in TNBC remains controversial, with some studies suggesting it may contribute to tumor progression in a subset of TNBC cases, while others show limited therapeutic benefit from targeting AR in these tumors [6].

In addition to AR, tumor-associated neutrophils (TANs) have garnered attention for their role in the breast cancer tumor microenvironment. TANs are known to be recruited to the tumor site in response to inflammatory signals and can promote tumor growth and metastasis [7]. While TANs have been linked to more aggressive tumor behavior, their impact on disease progression and their interactions with other tumor markers like AR are not fully understood.

Despite extensive research, the exact roles of AR and TANs in breast cancer remain unclear, and results are often conflicting, particularly regarding their influence on tumor aggressiveness, response to therapy, and overall prognosis [8]. The interplay between these two markers and their combined impact on tumor differentiation, vascular invasion, and disease stage has not been well characterized, leaving a gap in our understanding of how they jointly contribute to breast cancer progression. Assessing these interactions provides deeper insight into tumor biology and microenvironmental dynamics.

Our study is aimed at addressing these gaps by evaluating the expression of AR and TANs across different breast cancer subtypes, grades, and stages. Specifically, we seek to clarify how AR and TAN expression patterns correlate with tumor invasiveness, differentiation, and other tumor biomarkers.

## 2. Methods

This retrospective cross-sectional study included female patients diagnosed with primary breast cancer at Stages I–IV who were referred to and had pathology-confirmed diagnoses at Borna Pathology Center in Zanjan Province, Iran, between April 2018 and March 2021. Patients were identified by reviewing pathology reports and medical records archived in the center's database. Only those with complete clinical data, histopathological slides, and confirmed staging according to the AJCC criteria were included. Exclusion criteria were incomplete records or uncertain staging. A total of 84 women met the inclusion criteria and were enrolled.

The study involved women who had undergone lumpectomy, mastectomy, or CNB (core needle biopsy), and whose tumor tissue samples were sufficient for pathological diagnosis and assessment of the markers under study. A total of 84 women were included. The samples were reviewed by a pathologist, and using the IHC (immunohistochemistry) method, TAN and AR were reported as either positive or negative. This was performed on existing tissue blocks, and grouping was done based on the positivity or negativity of the receptors (PR, ER, and HER2) and staging (four groups).

The following is the grouping based on receptors:

• HER2±/ER+/PR+ (Luminal A, B)
• HER2+/ER−/PR−
• HER2−/ER−/PR− (triple negative)

The study was approved by the Ethics Committee of Zanjan University of Medical Sciences, and written informed consent was obtained from all participants (ethical number: A-11-134-13). All data were anonymized prior to analysis to ensure patient confidentiality.

### 2.1. Tissue Preparation and Biomarker Detection

IHC was performed on formalin-fixed, paraffin-embedded tissue sections using the following primary antibodies: ER (clone SBC-1020), PR (clone SBC-1019), HER2 (clone SBC-972), and Ki-67 (clone SBC-973), all procured from Long Island Antibody, and AR (clone C1B4) obtained from CELNOVTE. Antibody dilutions were optimized according to manufacturer recommendations and laboratory protocols: ER, PR, HER2, and Ki-67 were used at dilutions ranging from 1:100 to 1:200, and AR was used at a dilution of 1:100–1:250.

For IHC analysis, 4-micron sections were prepared from formalin-fixed, paraffin-embedded tumor samples. After deparaffinization, rehydration, and antigen retrieval, primary antibodies for ER, PR, HER2, AR, and Ki67 were applied. Stained slides were reviewed by two pathologists under a microscope.

ER, PR, and AR were reported as positive or negative based on standard thresholds. AR, ER, and PR were considered positive if ≥ 1% nuclear staining was observed [9]. HER2 scoring followed standard ASCO/CAP guidelines [10]. HER2 expression was scored from 0 to 3+: scores of 0 or 1+ were considered negative, 3+ positive, and 2+ equivocal, requiring further confirmation via fluorescence in situ hybridization (FISH) to assess gene amplification. Ki67 was positive if ≥ 20% of tumor cells were stained [11].

### 2.2. Neural and Vascular Invasion Detection

Neural invasion (perineural invasion) and vascular invasion (lymphovascular invasion) were assessed using routine hematoxylin and eosin (H&E) staining. Neural invasion was defined by the presence of tumor cells around or within nerve fibers, while vascular invasion was identified by tumor cells within blood vessels or lymphatic channels.

### 2.3. TAN Methodology

TAN positivity was scored on H&E-stained sections by identifying neutrophils adjacent to tumor cells across 10 nonoverlapping high-power fields (40x magnification). Fields were selected from areas with the highest neutrophil density, avoiding necrotic regions and intravascular neutrophils. A case was considered TAN-positive if more than one neutrophil was observed in contact with tumor tissue in at least 10 fields. Scoring was performed independently by two blinded pathologists.

### 2.4. Data Collection

Patient data, including age, Ki67, PR, HER2, ER, grade, neural invasion, vascular invasion, TAN, AR, and stage, were recorded using a structured checklist for further analysis. All assessments, including IHC staining, TAN evaluation, and the detection of neural and vascular invasion, were performed by an expert pathologist.

### 2.5. Statistical Analysis

The data, after being recorded in the checklist, were entered into SPSS software Version 26 for analysis. Qualitative variables were reported as frequency (percentage) and quantitative variables as mean (standard deviation). The chi-square test (or Fisher's exact test) was used to evaluate the relationship between TAN and AR with pathological findings. Additionally, the relationship between patients' age and TAN/AR was assessed using an independent *t*-test. To evaluate the combined simultaneous effect of pathological findings, age, and disease stage on TAN and AR, a two-level logistic regression model was used. Moreover, the relationships in between five subtypes (defended by ER, PR, HER2, and Ki67), stage, age, metastasis (M), and grade with AR and TAN were studied separately and simultaneously using univariate logistic regression. A significance level of 0.05 was considered in all analyses. To account for multiple comparisons, Bonferroni correction was applied, adjusting the significance threshold by dividing the standard alpha (0.05) by the number of tests performed.

## 3. Results

### 3.1. Frequencies of Breast Cancer Stage, Grade, and Markers

Data from 84 patients were evaluated, with a mean age of 49.35 ± 11.15 years. The majority had T2 tumors (55.4%), N1 lymph node involvement (33.8%), and M0 (69.2%). Most tumors were moderately differentiated (65.8%), and the majority were ER-positive (77.4%), PR-positive (63.1%), and HER2-negative (82.1%). Ki67 was positive in 69.0% of cases, AR in 70.2%, and TAN in 45.6%. Vascular invasion was observed in 50.8%, while neural invasion occurred in 32.3%. Further details regarding the frequencies of breast cancer stage, grade, and markers are provided in Table 1.

### 3.2. Comparison of Age at Diagnosis With AR and TAN

There was no significant difference in age at diagnosis for AR or TAN expression. The mean ages were nearly identical for AR-positive (49.25) and AR-negative (49.56) patients (*p* = 0.91), as well as for TAN-positive (43.89) and TAN-negative (49.33) patients (*p* = 0.86) (Table 2). No statistically significant differences were observed in age at diagnosis between AR-positive and AR-negative patients or TAN-positive and TAN-negative patients, even after Bonferroni correction (adjusted *α* = 0.025).

### 3.3. AR Expression

AR positivity was highest in Luminal A and Luminal B subtypes (82%). Similarly, AR positivity was high among HER2-positive patients (85%), further indicating its relevance in this subtype. In contrast, AR positivity was much lower in TNBC, with only 20% of patients testing positive for AR (OR: 0.06, 95% CI: 0.01–0.3), and this association remained significant after Bonferroni correction (Table 3).

Regarding disease stages, AR positivity was similar between early (Stages I/II) and late-stage breast cancer (Stages III/IV), with 71.4% and 69.0%, respectively, expressing AR, suggesting that AR expression does not vary significantly with cancer stage (OR: 0.9, 95% CI: 0.3–2.5). Tumor grade, however, was significantly associated with AR expression and persisted as significant after Bonferroni correction. AR positivity was found in 80% of well-differentiated tumors, but only 29% of poorly differentiated tumors expressed AR (OR: 0.1, 95% CI: 0–0.7), suggesting that AR is more frequently expressed in less aggressive, well-differentiated tumors (Table 3).

AR expression was strongly associated with ER positivity, with 83.1% of ER-positive cases also being AR-positive (*p* < 0.001), highlighting a robust link between AR- and ER-positive breast cancers that remained statistically significant after Bonferroni correction (Table 4). Logistic regression further demonstrated that AR-positive tumors were over 74 times more likely to be ER-positive than AR-negative ones (OR = 74.31, *p* < 0.001). Additionally, AR positivity was significantly associated with PR positivity (86.8%, *p* < 0.001). AR expression was also higher in Ki67-negative tumors (80.8%) compared to Ki67-positive tumors (65.5%), though this difference was not statistically significant (*p* = 0.158) (Table 4).

No statistically significant associations were observed between AR or TAN expression and HER2, Ki67, neural invasion, or vascular invasion after correction, though PR status showed a marginal association with TAN (*p* = 0.044) prior to adjustment.

AR positivity was higher in well-differentiated tumors (80%, *p* = 0.035), while moderately (71.2%) and poorly differentiated tumors (28.6%) had lower AR expression, though these differences were not statistically significant (Table 5). Although tumor grade showed a notable association with AR expression, this did not meet the Bonferroni-adjusted threshold for statistical significance.

AR positivity was higher in tumors with vascular invasion (81.8% vs. 62.5%), and logistic regression confirmed a significant link between AR positivity and vascular invasion (OR = 9.04, *p* = 0.047), indicating that AR-positive tumors were nine times more likely to exhibit vascular invasion compared to AR-negative ones. While the association did not remain statistically significant after Bonferroni adjustment, a suggestive trend was still observed (Table 4).

### 3.4. TAN Expression

TAN expression was highest in TNBC, with 67% of patients showing TAN positivity (OR: 3.7, 95% CI: 0.9–15.3), indicating that TAN may play a role in this aggressive subtype, but this association was attenuated following Bonferroni correction. In contrast, TAN positivity was lower in Luminal A (35%), Luminal B (47%), and HER2-positive (38%) subtypes, suggesting that TAN expression is less common in HR+ and HER2-driven cancers.

TAN positivity was slightly more frequent in late-stage disease (44.4%) compared to early-stage disease (41%), but this difference was not statistically significant (OR: 1.2, 95% CI: 0.4–3.1). TAN expression was highest in poorly differentiated tumors, with 71.4% showing TAN positivity (OR: 2.2, 95% CI: 0.3–14.8), suggesting that TAN may be more prevalent in aggressive, poorly differentiated tumors, although the association was not statistically significant (Table 5).

TAN positivity was significantly higher in PR-negative cases (60.0%, *p* = 0.044), indicating that TAN may play a larger role in PR-negative breast cancers (Table 4). Logistic regression further supported this inverse relationship, with TAN positivity being significantly less likely in PR-positive tumors (OR = 0.21, *p* = 0.014) (Table 6).

Neither AR nor TAN expression was significantly associated with metastatic status (Table 5). No significant association was found between AR or TAN expression and HER2 status (*p* = 0.536 for AR, *p* = 0.630 for TAN), as AR and TAN positivity rates were similar between HER2-positive and HER2-negative cases (Table 4). Similarly, there was no significant association between TAN expression and neural invasion (*p* = 0.913 for AR and *p* = 0.212 for TAN).

Simultaneous AR and TAN positivity was highest in Luminal B (37%) and lowest in TNBC (13%), occurring more often in early-stage (30.8%) and well-differentiated tumors (41%) than in late-stage (18.5%) and poorly differentiated ones (14%) (Table 3).

## 4. Discussion

AR positivity is significantly predictive of HR status, making it a potential marker for identifying hormone-driven tumors. This finding suggests that AR could be utilized to refine patient stratification and guide therapeutic decisions, particularly in ER-positive breast cancers. Several studies have highlighted the role of AR in ER- and PR-positive breast tissues. For instance, Tsang et al. [12] found a significant correlation between AR and PR positivity, indicating that AR can serve as both a prognostic marker and a therapeutic target. Similarly, Qi et al. [13] reported that AR expression was strongly associated with ER and PR positivity, underscoring its relevance in specific subtypes of breast cancer [14].

Additionally, our data revealed that AR positivity was more prevalent in well-differentiated tumor types. This suggests that AR expression may be linked to a less aggressive tumor phenotype and could serve as an indicator of tumor differentiation status. Supporting this, Hickey et al. [15] emphasized the importance of AR expression in ER-positive breast cancers, where AR acts as a tumor suppressor by directly inhibiting ER-driven transcriptional activity. Its activation counteracts the pro-proliferative effects of ER signaling, thereby reducing cell proliferation and improving prognosis. Rahim and O'Regan, in their review, concluded that AR signaling is associated with a better prognosis in ER-positive tumors [16]. Likewise, Park et al. found that AR-positive tumors tend to have lower histologic grades, smaller tumor sizes, and better differentiation [17]. Moreover, specific AR variants have been identified as potential predictors of breast cancer aggressiveness.

Further corroborating these findings, Niemeier et al. [18] observed that AR was expressed in 80% of all breast cancers, with 95% of ER-positive tumors also showing AR positivity. In ER-positive cases, AR expression was linked to smaller tumor sizes, lower Nottingham grades, and less frequent tumor necrosis. Castellano et al. [19] provided additional evidence that AR expression not only marks a more benign tumor profile but also serves as an independent prognostic factor for better survival outcomes in ER-positive breast cancers. Similarly, Yu et al. [20] demonstrated that AR expression is more frequently observed in Luminal A and B subtypes compared to basal-like subtypes and that AR-positive tumors showed a lower incidence of relapse and distant metastasis.

However, our study, along with others, indicates that AR's role in TNBC is limited. Gasparini et al. [21] reported that AR is expressed in only 24.8% of TNBC cases compared to 81.6% in non-TNBC cases, with AR presence being more prominent in nonbasal TNBC subtypes. This limited expression reduces AR's utility as a prognostic or therapeutic marker in TNBC. Shah et al. [22] also noted a lack of substantial therapeutic impact of AR-targeted therapies in AR-positive TNBC patients, reinforcing the notion that AR's role in TNBC is minor and confined to a small subset of tumors. As such, AR status is unlikely to significantly influence prognosis or therapeutic strategies for the broader TNBC population.

Interestingly, our results also showed that tumors with vascular invasion were more likely to be AR-positive. This complex role of AR, being linked to both less aggressive histology and increased invasiveness, has been noted in several previous studies [23–25]. These findings suggest that AR may not serve as a straightforward marker of tumor behavior, emphasizing the need for a nuanced interpretation of AR status. Context-specific factors likely modulate AR's role in breast cancer, as existing studies suggest AR's behavior is highly dependent on the surrounding tumor environment and associated oncogenic signals [4, 26].

We found that TANs were significantly more prevalent in TNBC and poorly differentiated tumors. This aligns with findings by Villareal-Garza et al. [27], who also reported the involvement of TANs in more aggressive breast cancers. Researchers have explored the underlying mechanisms, attributing the presence of TANs in TNBC to their biological significance within the tumor microenvironment. Martins-Cardoso et al. [7] demonstrated that neutrophil extracellular traps (NETs) released by TANs promote a prometastatic phenotype in breast cancer by driving epithelial-to-mesenchymal transition and enhancing cell migration. This supports our observation that TANs are associated with more aggressive tumor characteristics.

Additionally, studies have shown that TANs are preferentially recruited by highly aggressive TNBC cells, contributing to increased metastasis and poor prognosis [28]. Hein et al. [29] reported that TAN migration is further enhanced by factors released from aggressive TNBC cells, suggesting a direct link between TANs and tumor invasiveness. Similarly, Chen et al. [30] showed that elevated PANX1 expression in TNBC cells drives neutrophil recruitment, reinforcing the role of TANs in aggressive disease phenotypes.

Given these findings, TANs could serve as valuable prognostic biomarkers for identifying more aggressive breast cancers. Furthermore, targeting TAN recruitment or activity could be a promising therapeutic strategy to mitigate their protumor effects in high-risk subtypes. The potential of blocking neutrophil recruitment pathways, such as the CXCR2 axis, in the treatment of aggressive breast cancers like TNBC is an area of active research [31–33]. While these strategies are currently under investigation in preclinical and early clinical studies, larger scale trials are needed to validate their clinical utility.

Regarding HR expression, an inverse relationship between PR expression and certain aggressive features of breast cancer has been reported in other studies as well [34, 35]. Gupta et al. [36] found a strong inverse correlation between PR expression and lymphomononuclear infiltrates, suggesting that PR-negative tumors may have a more proinflammatory profile. Furthermore, loss of PR expression has been linked to more aggressive tumor subtypes [37].

In contrast, we found no significant associations between AR or TAN expression and metastatic status or perineural invasion. This indicates that these markers may not be reliable predictors of disease spread to distant organs, thereby diminishing their utility as prognostic tools for assessing metastasis risk or guiding systemic therapy decisions aimed at controlling or preventing metastatic disease. Instead, it is possible that these markers primarily influence the local tumor microenvironment rather than facilitating distant spread or neural invasion. Therefore, the clinical relevance of AR and TAN may be more confined to specific contexts, such as early-stage disease or localized tumor behavior.

One of the main limitations of our study is the small sample size of 84 patients, which may have reduced the statistical power to detect subtle associations, especially in less common subtypes like TNBC. Additionally, the cross-sectional design prevents us from drawing causal inferences or assessing temporal changes in AR and TAN expression over time. Due to the retrospective nature of the study, disease-free and overall survival data were not available, which limited our ability to evaluate the prognostic significance of the biomarkers studied. The lack of functional validation further limits our ability to confirm whether AR and TAN actively influence tumor differentiation, invasiveness, and vascular involvement. These findings are intended to generate hypotheses and inform future research. Future studies with larger cohorts, long-term follow-up, and experimental validation are needed to address these gaps.

## 5. Conclusion

Our study highlights that AR expression is significantly associated with HR+ breast cancers and better differentiation, making it a valuable marker for patient stratification. In contrast, TANs are linked to more aggressive phenotypes, particularly in TNBC, suggesting a role in tumor progression. However, the absence of associations with metastatic status and neural invasion limits their utility in assessing these specific aspects of disease progression.

## Figures and Tables

**Table 1:** Frequencies of breast cancer stage, grade, and markers.

**Stage/grade**	**Category**	**Count (%)**
T stage	T1	18 (27.7%)
	T2	36 (55.4%)
	T3	4 (6.2%)
	T4	7 (10.8%)

N stage	Nx	1 (1.5%)
	N0	17 (26.2%)
	N1	22 (33.8%)
	N2	12 (18.5%)
	N3	13 (20.0%)

M stage	M0	45 (69.2%)
	M1	20 (30.8%)

Grade	Well-differentiated	20 (25.3%)
	Moderately differentiated	52 (65.8%)
	Poorly differentiated	7 (8.9%)

Marker	Negative (%)	Positive (%)

ER	19 (22.6%)	65 (77.4%)

PR	31 (36.9%)	53 (63.1%)

HER2	69 (82.1%)	15 (17.9%)

Ki67	26 (31.0%)	58 (69.0%)

Neural invasion	44 (67.7%)	21 (32.3%)

Vascular invasion	32 (49.2%)	33 (50.8%)

AR	25 (29.8%)	59 (70.2%)

TAN	43 (54.4%)	36 (45.6%)

Abbreviations: AR, androgen receptor; ER, estrogen receptor; HER2, human epidermal growth factor receptor 2; PR, progesterone receptor; TAN, tumor-associated neutrophils.

**Table 2:** Comparison of age at diagnosis with AR and TAN.

**Variable**	**Negative mean (SD)**	**Positive mean (SD)**	**Test statistic**	**p** ** value**
AR	49.56 (13.01)	49.25 (10.39)	0.11	0.91
TAN	49.33 (10.51)	43.89 (12.30)	0.17	0.86

Abbreviations: AR, androgen receptor; TAN, tumor-associated neutrophil.

**Table 3:** Logistic regression results of androgen receptor (AR) and tumor-associated neutrophils (TAN) with breast cancer subtypes, stages, and pathology markers.

**Variable category**	**AR+** **N** ** (%)**	**AR+** **OR (95% CI)**	**TAN+** **N** ** (%)**	**TAN+** **OR (95% CI)**	**AR+ and TAN+** **N** ** (%)**	**AR+ and TAN+** **OR (95% CI)**	**AR− and TAN−** **N** ** (%)**
Subtype							
Luminal A	18 (82%)		7 (35%)		6 (30%)		2 (10%)
Luminal B	27 (82%)	1.0 (0.2, 4.0)	14 (47%)	1.6 (0.5, 5.2)	11 (37%)	1.3 (0.4, 4.5)	2 (7%)
HER2+	11 (85%)	1.2 (0.2, 7.8)	5 (38%)	1.2 (0.3, 4.9)	3 (23%)	0.6 (0.2, 3.0)	0
Triple negative	3 (20%)	0.06 (0.01, 0.3)	10 (67%)	3.7 (0.9, 15.3)	2 (13%)	0.4 (0.1, 2.7)	4 (27%)
Stage							
I/II	30 (71.4%)		16 (41.0%)		12 (30.8%)		6 (15.4%)
III/IV	20 (69.0%)	0.9 (0.3, 2.5)	12 (44.4%)	1.2 (0.4, 3.1)	5 (18.5%)	0.4 (0.1, 1.5)	2 (7.4%)
Grade							
Well-differentiated	16 (80%)		9 (53%)		7 (41%)		1 (6%)
Moderately differentiated	37 (72%)	0.7 (0.2, 2.3)	21 (41%)	0.6 (0.2, 1.9)	13 (25%)	0.5 (0.2, 1.7)	6 (12%)
Poorly differentiated	2 (29%)	0.1 (0, 0.7)	5 (71%)	2.2 (0.3, 14.8)	1 (14%)	0.2 (0, 2.7)	1 (14%)

Abbreviations: AR, androgen receptor; HER2, human epidermal growth factor receptor 2; TAN, tumor-associated neutrophils.

**Table 4:** The association of AR and TAN with ER, PR, Her2, Ki67, neural invasion, and vascular invasion variables.

**Variable**	**Category**	**AR**				**TAN**			
		**Negative, ** **N** ** (%)**	**Positive, ** **N** ** (%)**	**Test statistic**	**p** ** value**	**Negative, ** **N** ** (%)**	**Positive, ** **N** ** (%)**	**Test statistic**	**p** ** value**
ER	Negative	14 (73.7)	5 (26.3)	22.66	< 0.001	7 (36.8)	12 (63.2)	3.12	0.077
	Positive	11 (16.9)	54 (83.1)			36 (60.0)	24 (40.0)		
PR	Negative	18 (58.1)	13 (41.9)	18.83	< 0.001	12 (40.0)	18 (60.0)	4.06	0.044
	Positive	7 (13.2)	46 (86.8)			31 (63.3)	18 (36.7)		
HER2	Negative	22 (31.9)	47 (68.1)	—	0.536	34 (53.1)	30 (46.9)	0.23	0.630
	Positive	3 (20.0)	12 (80.0)			9 (60.0)	6 (40.0)		
Ki67	Negative	5 (19.2)	21 (80.8)	2.00	0.158	16 (66.7)	8 (33.3)	2.08	0.149
	Positive	20 (34.5)	38 (65.5)			27 (49.0)	28 (51.0)		
Neural invasion	Negative	12 (27.3)	32 (72.7)	0.01	0.913	22 (55.0)	18 (45.0)	1.56	0.212
	Positive	6 (28.6)	15 (71.4)			15 (71.4)	6 (28.6)		
Vascular invasion	Negative	12 (37.5)	20 (62.5)	3.03	0.082	16 (55.2)	13 (44.8)	0.70	0.404
	Positive	6 (18.2)	27 (81.8)			21 (65.6)	11 (34.4)		

Abbreviations: AR, androgen receptor; ER, estrogen receptor; HER2, human epidermal growth factor receptor 2; PR, progesterone receptor; TAN, tumor-associated neutrophils.

**Table 5:** Association of AR and TAN with breast cancer grade and stages.

**Variable**	**Category**	**AR**			**TAN**		
		**Positive, ** **N** ** (%)**	**Test statistic**	**p** ** value**	**Positive, ** **N** ** (%)**	**Test statistic**	**p** ** value**
Grade	Well-differentiated	16 (80)	6.65	0.035	9 (52.9)	2.81	0.253
	Moderately differentiated	37 (71.2)			21 (40.4)		
	Poorly differentiated	2 (28.6)			5 (71.4)		

T stage	T1	12 (66.7)	0.28	0.810	5 (29.4)	0.001	0.807
	T2	28 (77.8)			13 (39.4)		
	T3	2 (50)			2 (50.0)		
	T4	5 (71.4)			4 (57.1)		

N stage	Nx	0%	2.18	0.239	4 (23.5)	0.09	0.989
	N0	10 (58.8)			4 (23.5)		
	N1	18 (81.8)			11 (52.4)		
	N2	11 (91.7)			3 (30.0)		
	N3	8 (61.5)			6 (46.2)		

M stage	M0	35 (77.8)	0.09	0.767	16 (38.1)	0.09	0.767
	M1	12 (60.0)			8 (42.1)		

Abbreviations: AR, androgen receptor; TAN, tumor-associated neutrophil.

**Table 6:** Logistic regression results for AR and TAN.

**Variable**	**Dependent variable**	**Regression coefficient**	**Standard error**	**p** ** value**	**OR (95% CI)**
ER-positive	AR	4.31	1.14	< 0.001	74.31 (7.99–691.54)
Vascular invasion-positive	AR	2.20	1.11	0.047	9.04 (1.03–79.30)
Age at diagnosis	TAN	− 0.06	0.03	0.064	0.94 (0.89–1.003)
PR-positive	TAN	− 1.56	0.63	0.014	0.21 (0.06–0.73)

Abbreviations: ER, estrogen receptor; PR, progesterone receptor.

## Data Availability

The datasets used and/or analyzed during the current study are available from the corresponding author on reasonable request.
